# Sheathless microcatheter: a new approach for musculoskeletal arterial embolization

**DOI:** 10.1186/s42155-026-00657-z

**Published:** 2026-02-07

**Authors:** Raad Madkhali, Mohammed Almoaiqel, Refaat Salman, Mohammad Arabi, Shaker Alshehri, Turki Alenazi, Elan Humoud, Abdulmohsen Alhussaini, Mohamed Rajab Elzahrani

**Affiliations:** 1 King Abdulaziz Medical City, Riyadh, Saudi Arabia; 2 King Abdullah International Medical Research Center, Riyadh, Saudi Arabia

## Abstract

**Introduction:**

Ongoing developments in interventional radiology have enabled the treatment of various musculoskeletal inflammatory disorders via super-selective embolization of abnormal areas of neovascularization. The current convention is to utilize vascular sheaths in these procedures. This study explored a single-center experience with a novel approach utilizing a sheathless microcatheter cannulation technique without the conventional vascular sheath.

**Material and methods:**

A single-center retrospective chart review was conducted, including all eligible adult patients for whom a super-selective embolization was performed to treat an MSK inflammatory disorder between August 2024 and March 2025. The pre-procedural medical records and imaging were reviewed to determine satisfaction of inclusion criteria. The fluoroscopic images from the angiography suite and procedure notes were reviewed to determine technical success and immediate complications.

**Results:**

Twenty-seven procedures were performed for 19 patients. The average age was 47.5 ± 10 years old. The majority of patients were female (13; 68.5%). The most common procedure indication was for treatment of plantar fasciitis with 14 procedures (51.9%). The embolic material of choice was imipenem-cilastatin in all but one procedure (26; 96.3%). The commonest microcatheter size was 1.7 French (21; 77.8%), and the preferred access vessel was the common femoral artery (17; 63%). The technical success rate was 100% without immediate complications. No major post-procedural complications were encountered. Only one minor access site hematoma complication was reported.

**Conclusion:**

This novel sheathless microcatheter approach was found to be feasible, safe, and effective without major complications within our limited sample size.

## Introduction

The use of interventional radiology (IR) in musculoskeletal (MSK) pathologies is increasing, both through embolotherapy and other various interventions [1]. Neovascularization and the accompanying neural ingrowth explain pain in various MSK pathologies [2, 3]. With advances in both imaging and radiologic interventions, the selective embolization of neo-vessels provides a treatment option for various MSK pathologies. For instance, genicular artery embolization is a procedure in which selective catheterization and embolization are performed targeting abnormal arterial blush supplying the knee joint’s synovium. While no studies demonstrated any disease-modifying effects, GAE is effective in decreasing pain and inflammation with a low retreatment rate [4]. Plantar fasciitis, while self-limiting, is a painful and often challenging condition to manage. A novel treatment for plantar fasciitis is selective embolization of the posterior tibial artery, which shows high rates of both technical success and pain relief in the limited literature [5]. Selective catheterization and embolization have been used in other MSK conditions such as adhesive capsulitis [6], lateral epicondylitis [7], and De Quervain’s tenosynovitis [8].

The various previously described MSK interventions are often performed with a vascular sheath and multiple catheter sizes, most often 4 French [5, 9]. The use of smaller catheter sizes has been associated with lower complication rates [10]. Therefore, sheathless microcatheter use was recently introduced in our center for vascular MSK interventions, and this study aims to assess the safety and feasibility of this new approach in the setting of MSK interventions.

## Material and methods

The study was performed as a single-center retrospective chart review and analysis of MSK vascular interventions in King Abdulaziz Medical City, Riyadh, Saudi Arabia; a tertiary referral center and level 1 trauma center. The study received institutional review board approval from King Abdullah International Medical Research Center (IRB number 00000138525) and informed consent was waived. The study included all musculoskeletal intervention cases for which a sheathless microcatheter technique was used for selective embolization to treat pain and inflammation (e.g., geniculate artery embolization) between August 2024 and March 2025. Any cases where a vascular sheath was used or patients under 14 years of age were excluded.

Procedural success was defined as selective embolization of the targeted vessels. To determine the procedure safety, complications were stratified using the Society of Interventional Radiology (SIR) adverse event classification system [11]. Procedural adverse events were determined through reviewing procedure notes, while post-procedural complications were determined by reviewing the subsequent hospital records, follow-up imaging studies, and radiology reports.

Patients were assessed in the interventional radiology clinic for trans-arterial microembolization (TAME) suitability. Most patients were referred to IR from the orthopedic surgery department, while others were referred from other specialties (e.g., plastic surgery referred De Quervain’s tenosynovitis patients) or through self-referral. All patients had prior magnetic resonance imaging studies performed to assess for inflammatory changes prior to the planned intervention. All patients undergoing embolization for musculoskeletal pain and inflammation starting August 1, 2024, were treated using the sheathless approach. Patients were followed up in the clinic at 1 week, 4 weeks, and 6 months following their procedures.

### Technique

All cases were performed with local anesthesia and no sedation. Under ultrasound guidance, arterial access is established using a 21-gauge needle and microwire. The targeted artery is punctured in antegrade fashion. A microcatheter ranging in size from 1.7 to 1.8 French is introduced over the wire to the target area, and angiography is performed with manual injection of 5 of iodine contrast to delineate vascular anatomy and to determine neovascularization. Subsequently, arteries with neovascularization are super-selected using the microcatheter. Embolic material is injected manually in 0.2-mL increments until blood flow stagnation. Hemostasis is established through manual compression for 10 min, and bed rest is encouraged for 2 h.

### Embolic material

Imipenem/cilastatin is the embolic material of choice in most patients. Imipenem is a beta-lactam antibiotic that, when suspended in iodine contrast, forms particles ranging in size between 10 and 70 µm; these particles cause a temporary embolic effect on smaller vessels [12]. It is mixed in a 1:1 ratio with cilastatin, which inhibits renal enzyme dehydropeptidase I, leading to decreased imipenem degradation and increasing its efficacy [13]. Imipenem/cilastatin was first described as an embolic agent by Aihara in 1999 in animal models [14]. Woodhams et al. described the use of imipenem/cilastatin for embolization of gastrointestinal bleeds in patients with neoplasia in 2013 [15]. The use of imipenem/cilastatin for musculoskeletal pain was described by Okuno et al. in 2013 for patients with enthesopathy and tendinopathy with good early results [16]. The embolic material is prepared by mixing 0.5 g of imipenem/cilastatin with 5–10 mL of iodine contrast by pumping the syringe for 10 s. It is then drawn into a 1-mL syringe and injected at 0.2-mL increments. The total injected volume varies based on the procedure and degree of neovascularization.

### Post-procedural follow-up

Patients were reassessed in the interventional radiology clinic 1 week, 4 weeks, and 6 months following the procedure. During the follow-up visits, the assessment was limited to clinical history and physical examination. Further imaging and laboratory studies were not routinely performed. The follow-up period of the study was up to 6 months from the date of intervention, and included notes from the interventional radiology department, other departments, nursing notes, and relevant imaging, if present.

### Sample case

**Fig. 1:**
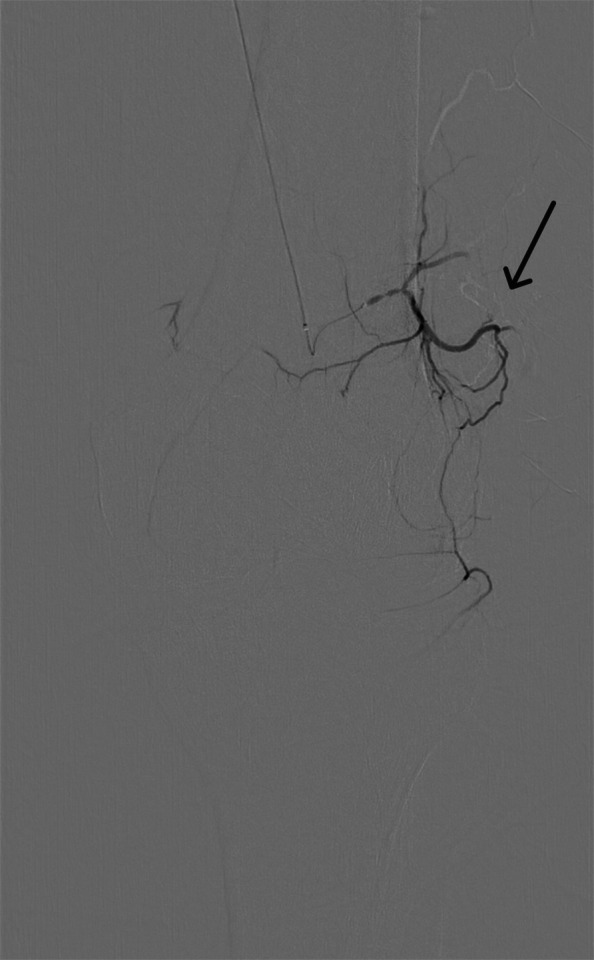
Lateral superior genicular artery angiogram

**Fig. 2:**
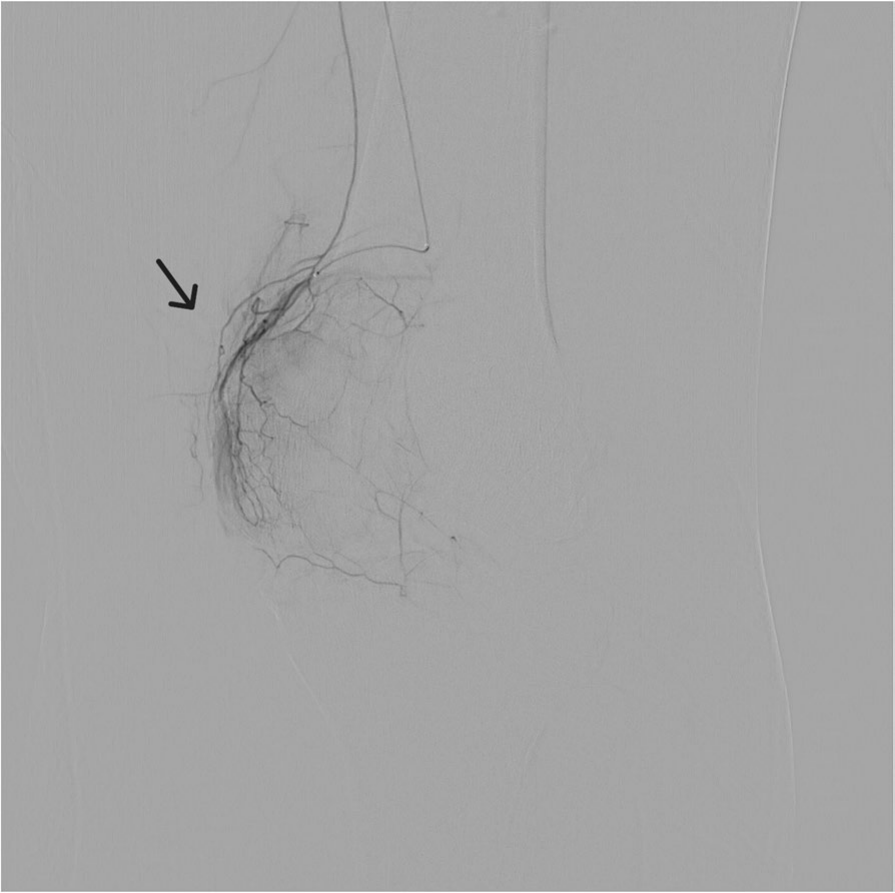
Medial superior genicular artery with a vascular blush apparent

**Fig. 3:**
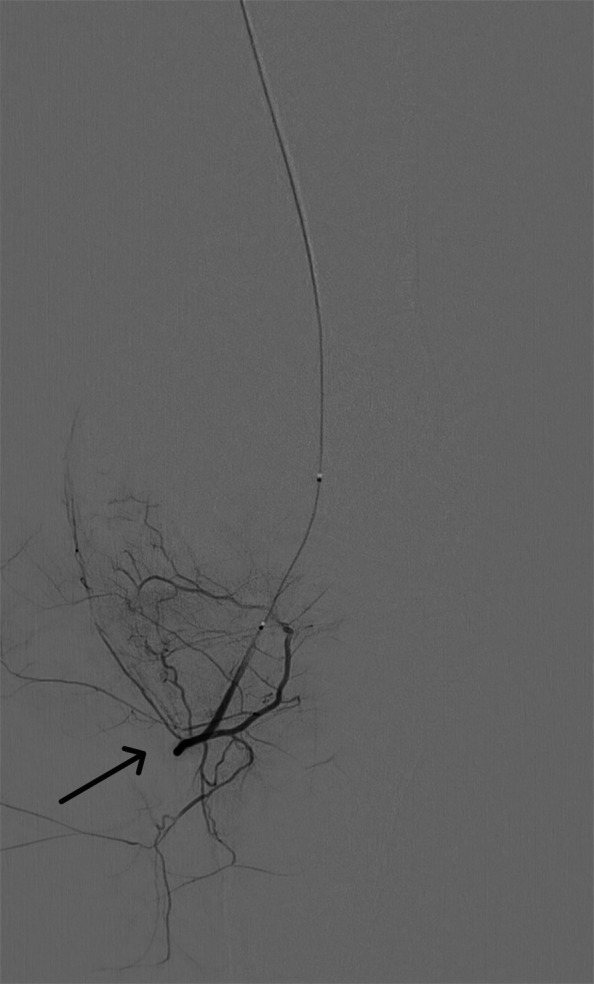
Medial inferior genicular artery with vascular blush

**Fig. 4:**
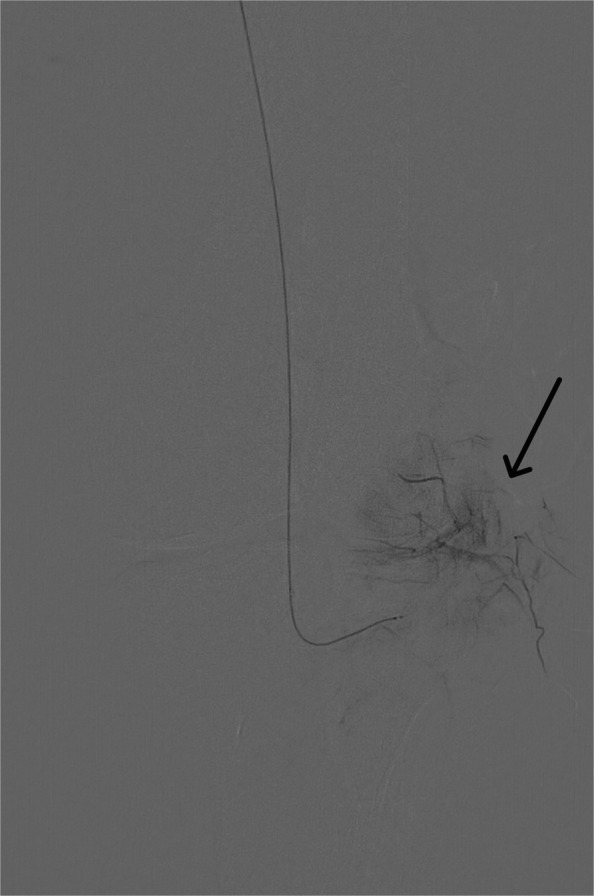
Lateral inferior genicular artery with abnormal vascular blush pre-embolization

**Fig. 5:**
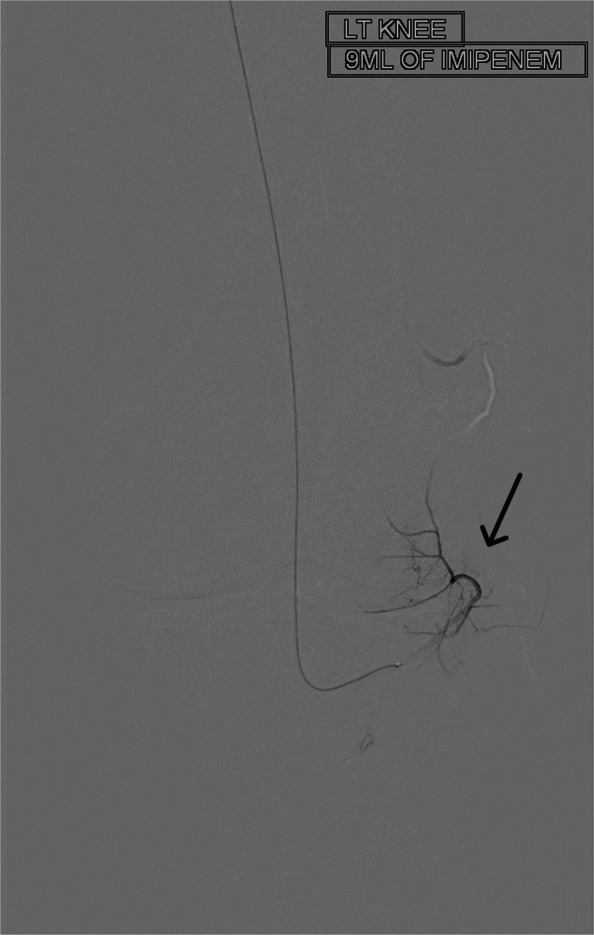
Lateral inferior genicular artery post-embolization with improvement of vascular blush

For this geniculate artery embolization procedure, ultrasound-guided access was established, through the common femoral artery, using a 21-gauge needle and microwire [Synchro2® (Stryker, Kalamazoo, MI, USA)], followed by introducing the microcatheter [Echelon 10 MicroCatheter (45- and 90-degree angle; Medtronic, Dublin, Ireland)]. The microcatheter is then advanced in an antegrade manner to the target vessels on the ipsilateral side. In this case, the target vessels were the medial and lateral superior and inferior geniculate arteries (Figs. 1, 2, 3, and 4). Subsequently, a diagnostic angiogram was performed at these vessels to assess for the vascular blush denoting neovascularization, as seen in Figs. 2, 3, and 4; these identified areas were then embolized using a mixture of imipenem and cilastatin suspended in iodine contrast material. In the sample case, the genicular vessels showed vascular blush and were embolized. A final angiogram was obtained following embolization to ensure the resolution of the vascular blush as seen in Fig. 5 in the lateral inferior genicular artery. A post-embolization angiogram was performed in all 4 vessels. The process is similar in other musculoskeletal embolotherapies with appropriate modifications to the access site and targeted vessels. Hemostasis was achieved through manual compression for 10 min. Bed rest was encouraged for 2 h, and the patient was discharged the same day.

### Data analysis

The variables collected included demographic variables (age and sex). The independent variables were the procedure type, access site and side, microcatheter size, targeted artery, embolic material used, and any complications. The data were analyzed using the statistical package for the social sciences (SPSS) version 26. Data are presented as percentages and frequency tables.

## Results

The study included 19 patients, for whom 27 procedures were performed. The majority were females (13; 68.5%). The mean age of patients was 47.5 ± 10 years old. Of the 27 procedures, the most common indication was plantar fasciitis with 14 procedures (51.9%) performed for 9 patients, followed by genicular artery embolization with 7 procedures (25.9%) for 5 patients. The remaining 6 procedures (22.2%) were performed for various miscellaneous indications (Table 1). The most commonly accessed vessel was the common femoral artery (17; 63%). The most common microcatheter size was 1.7 French (21; 77.8%) with a size range of 1.7–1.8 French. Imipenem-cilastatin was the embolic material of choice in all cases (26; 96.3%) aside from one case in which doxorubicin beads were used. The case in which doxorubicin beads were used was a female patient complaining of aggressive plantar fibromatosis for whom resection was attempted unsuccessfully in another hospital. In our institute, the patient underwent multiple treatments, including radiotherapy and systemic therapy, without success. The decision was made to proceed with targeted chemoembolization with doxorubicin beads. The technical variables are discussed in Table 2.

**Table 1:** Procedure indications

Procedure indication	*n*	%
Planter fasciitis	14	51.9
Knee osteoarthritis	7	25.9
De Quervain’s	1	3.7
Scaphoid fracture causing chronic pain	1	3.7
Achilles tendinosis	2	7.4
Ankle fibromatosis	1	3.7
Ankle tenosynovitis	1	3.7

**Table 2:** Technical procedural variables

Access site	*N* = 27	Percentage
Common femoral artery	17	63%
Superficial femoral artery	8	29.6%
Brachial artery	2	7.4%
Access side		
Right	14	51.9%
Left	13	48.1%
Targeted artery		
Genicular artery	7	25.9%
Posterior tibial artery	17	63%
Radial artery	2	7.4%
Peroneal artery	1	3.7%
Embolic material		
Imipenem-cilastatin	26	96.3%
Doxorubicin-eluting beads	1	3.7%

There were no procedural complications with a technical success rate of 100%. During post-procedural follow-up, only one minor complication was identified, which was an access site hematoma identified during a routine follow-up visit, which required no further intervention or admission for observation. The patient underwent super-selective embolization of the posterior tibial artery for treatment of plantar fasciitis. The access site was the common femoral artery, and post-procedural hemostasis was achieved by manual compression for 5 min. No special precautions following the procedure were taken, as this minor complication was detected during a follow-up visit 1 week after the procedure.

## Discussion

The role of interventional radiology in musculoskeletal (MSK) pathologies continues to expand, particularly through embolotherapy and other minimally invasive vascular interventions [1]. These techniques have shown promising results in managing MSK pain and inflammation, offering a less invasive alternative to traditional treatment methods. Conventionally, MSK embolization procedures are performed using vascular sheaths, most commonly 3–5-French catheters. However, the use of smaller, sheathless catheters has been associated with lower complication rates in various vascular interventions [10]. The sheathless microcatheter technique was developed to further enhance procedural safety and efficacy by reducing trauma to vascular structures while maintaining high success rates in MSK interventions. Given the increasing adoption of embolization for MSK conditions such as osteoarthritis, plantar fasciitis, and tendinopathies, refining vascular access techniques may further optimize patient outcomes and minimize procedural risks.

Complications associated with MSK embolization can be divided into procedural and post-procedural complications. Procedural complications include microartery perforation, arterial dissection, and microcatheter tip fractures and other hardware complications [17]. Post-procedural complications vary in their severity and time of onset. This includes complications such as access site hematomas and hemorrhages [18], transient skin discoloration [19], and puncture site pain [20], and paresthesia relating to non-target embolization [17].

Ischemia-related complications can be potentially dangerous; this includes tissue infarctions (e.g., bone infarction) and tendon rupture [16, 17]. Ischemia-related complications, as well as paresthesia, have been attributed to non-target embolization [21]. This type of complication has been associated with smaller embolic material particle sizes (< 100 µm), as seen in both human and animal studies [22, 23]; this led Bagla et al. [22] to use larger particle sizes (> 100 µm) following 2 cases of plantar paresthesia believed to be due to non-target embolization of the medial plantar nerve. Although the embolic material of choice in our study, imipenem/cilastatin, has a particle size of < 100 µm, it has a transient effect due to its water solubility [23].

The most common indication for MSK interventions in the study was plantar fasciitis. Plantar fasciitis is the most common cause of inferior heel pain, accounting for 80% of cases [24]. It is a multifactorial degenerative process of micro-tears with an estimated international prevalence rate of 3.6 to 7% [25]. Plantar fasciitis may be treated conservatively with rest, stretching, physical therapy, orthoses, extracorporeal shockwave therapy, or laser therapy [26], while refractory cases may benefit from surgical management [27]. Transcatheter arterial embolization is a less invasive treatment option for patients with refractory plantar fasciitis. Gandhi and Banker reported a 100% technical success rate using 4-French catheters in 10 patients (13 procedures), with no significant complications or adverse effects [5]; this shows results similar to our study, in which 14 microembolizations were performed for plantar fasciitis with 100% technical success rate and no major complications.

The second most common indication for embolization in the sample population was osteoarthritis of the knee. Knee osteoarthritis is the most common form of arthritis [28], with a 45% lifetime risk of developing knee osteoarthritis. The curative treatment of choice for patients with severe osteoarthritis is total knee replacement. However, mild and moderate cases can be challenging to manage. The use of imipenem-cilastatin to selectively embolize the geniculate arteries has been described in the literature with satisfactory outcomes and high technical success rates. A study by Okuno et al. assessing the midterm outcomes of GAE in 72 patients (95 knees) showed 100% success rate and no major complications [19]. Another study by Okuno et al., utilizing a 3-French catheter for GAE in 14 patients, demonstrated a 100% technical success rate, with no major adverse events. The only reported complication was a moderate subcutaneous hemorrhage at the puncture site, which resolved within 1 week [12]. Lee et al. reported the clinical outcomes of GAE in a retrospective study involving 45 patients (78 knees) with 100% technical success and no major complications as well [29]. Little et al. examined GAE using a 4-French vascular sheath in 38 patients, reporting a technical success rate of 84%. Their study noted four cases (12.5%) of mild, self-limiting skin discoloration due to non-target embolization and one patient with a small, self-limiting groin hematoma [9]. These studies show high technical success rates with no major complications and few minor complications. Seven patients underwent GAE using the sheathless technique with 100% technical success rate and no major adverse events, showing findings similar to the established literature.

In the sample size of 27 interventions, the sheathless microcatheter technique achieved a 100% technical success rate, with no major periprocedural complications and only one minor complication (a small hematoma) identified during a follow-up visit 1 week after the procedure. These results are similar to previous research, including a meta-analysis by Taslakian et al. [4], which reported a 99.7% technical success rate for genicular artery embolization (GAE) across multiple studies. A systematic review of trans-arterial embolization for the treatment of musculoskeletal pain, which included 7 articles and 4 abstracts, reported similar results with 100% technical success rate and no major complications [30]. In this study, a 100% technical success rate and lack of major complications were achieved with the advantage of not requiring a vascular sheath. This represents similar results when compared to the previously discussed studies in which a vascular sheath was used.

Our study was limited by the single-center, retrospective design and the small sample size. The lack of a control group further limits the findings. Larger, multi-center studies with longer follow-up duration are required to validate these results and provide a more comprehensive comparison between sheathless and sheath-based techniques.

## Conclusion

Within the limited sample size, the use of a sheathless technique in this single-center study was found to be a safe and effective option for a wide variety of musculoskeletal vascular interventions without major complications and one minor complication.

## Data Availability

The datasets used and/or analyzed during the current study are available from the corresponding author on reasonable request.

## Declarations

Ethics approval and consent to participate.

All procedures performed in studies involving human participants were in accordance with the ethical standards of the institutional and/or national research committee and with the 1964 Helsinki Declaration and its later amendments or comparable ethical standards. For this type of study, formal consent is not required. An institutional review board approval was acquired (IRB number 00000138525).
